# Research Progress and Future Trends of Low Temperature Plasma Application in Food Industry: A Review

**DOI:** 10.3390/molecules28124714

**Published:** 2023-06-12

**Authors:** Jiacheng Zhang, Qijing Du, Yongxin Yang, Jing Zhang, Rongwei Han, Jun Wang

**Affiliations:** 1 College of Food Science and Engineering, Qingdao Agricultural University, Qingdao 266109, Chinajingjingnihao_2001@163.com (J.Z.); 2 Qingdao Special Food Research Institute, Qingdao 266109, China

**Keywords:** food industry, sterilization, low temperature plasma, decontamination

## Abstract

Food nutrition, function, sensory quality and safety became major concerns to the food industry. As a novel technology application in food industry, low temperature plasma was commonly used in the sterilization of heat sensitive materials and is now widely used. This review provides a detailed study of the latest advancements and applications of plasma technology in the food industry, especially the sterilization field; influencing factors and the latest research progress in recent years are outlined and upgraded. It explores the parameters that influence its efficiency and effectiveness in the sterilization process. Further research trends include optimizing plasma parameters for different food types, investigating the effects on nutritional quality and sensory attributes, understanding microbial inactivation mechanisms, and developing efficient and scalable plasma-based sterilization systems. Additionally, there is growing interest in assessing the overall quality and safety of processed foods and evaluating the environmental sustainability of plasma technology. The present paper highlights recent developments and provides new perspectives for the application of low temperature plasma in various areas, especially sterilization field of the food industry. Low temperature plasma holds great promise for the food industry’s sterilization needs. Further research and technological advancements are required to fully harness its potential and ensure safe implementation across various food sectors.

## 1. Introduction

**Figure 1:**
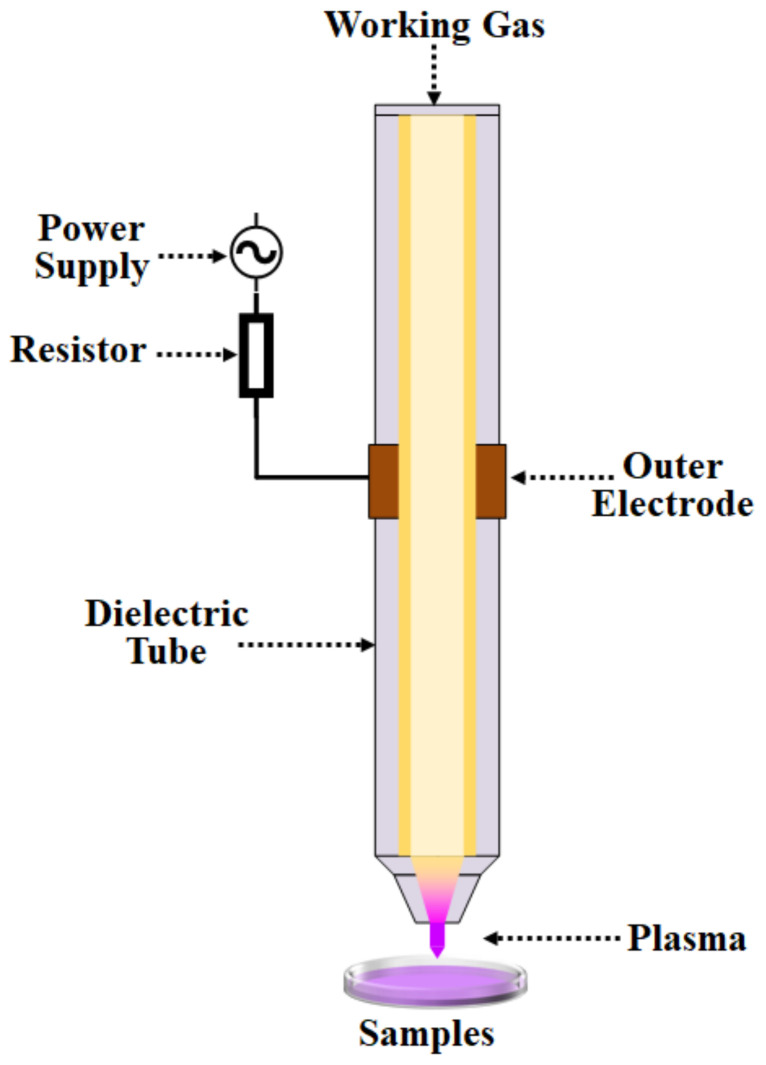
Principle of plasma processes.

In order to ensure microbial safety and quality and extend the shelf life of food, various sterilization technologies were applied in food industries. Traditional food sterilization methods mainly are thermal treatments [1]. The heating sterilization is an effective way to kill the microorganisms in the food at high temperature for a certain period. However, it often leads to a decrease in sensory quality and the loss of nutrients in the food, especially for fresh foods and heat-sensitive foods. Therefore, nonthermal sterilization techniques, including ultra-high pressure, radiation, ultrasound, ozone, pulsed strong light, UV treatment, pulsed electric field, electrolyzed water, and low temperature plasma and its activated water, etc., were widely studied in recent years [2,3,4,5,6,7,8]. Plasma, known as the fourth state of matter, is defined as a fully or partially ionized gas (Figure 1). It comprises both thermal and non-thermal plasma [9]. Non-thermal plasma, often referred to as low-temperature plasma, is a cutting-edge sterilization technique initially utilized in the printing and materials industry [9,10]. Recently, it emerged as a research hotspot in the field of food processing.

For more than 20 years, atmospheric low-temperature plasma was applied to food sterilization [11,12,13,14,15,16,17]. Irving Langmuir first used the term “plasma” in 1928 to define the fourth state of matter, that is, the partially or fully ionized state of matter, and discovered that the plasma oscillates in ionized gas [18]. Plasma is the fourth state of matter. It is an ionized gas containing a series of active substances such as electrons, free radicals, and ions [19], which is a state of matter that is electrically neutral as a whole and exists widely in the universe.

According to the type of energy supply and the amount of energy transferred to the plasma, plasma is divided into high-temperature plasma and low-temperature plasma [20]. In food sterilization, the most used technique is low temperature plasma, the main advantages of which include: (1) it can be adjusted freely, and heat-sensitive food can be sterilized at normal temperature; (2) the required sterilization time is short and non-toxic, only a few seconds or a few minutes; (3) sterilization in a closed system, the radiation generated by sterilization is not leaked; (4) the operation is simple and easy to use, and the food can be sterilized from various angles; (5) it helps extend the shelf life of food products, reducing food waste, and diminishes the reliance on chemical preservatives, offering a more natural and healthier food preservation method; (6) it finds broad applications in different sectors of the food industry, with ongoing rapid development. Several previous good reviews were published to summarize the principles of plasma technology, applications in the food industry including dairy products processing, food sterilization, packaging material processing, functionality modification of food materials and dissipation of agrochemical residues, and proposed prospects [19,21]. While low temperature plasma was extensively studied and offers numerous advantages in the field of food processing, it is important to recognize certain limitations associated with its application in the food industry. If the thickness of the food is large, the sterilization effect on deep bacteria is not good, mainly because the plasma penetration is not good enough. Although it has a good bactericidal effect on the surface of food [22], if the factors such as processing time are not well controlled, the physical and chemical indicators of food such as color and pH will also change. Due to the rapid development of plasma technology, researchers from various countries carried out many studies. This paper aimed to review and update the application of plasma technology in the food industry, especially sterilization, including influencing factors and the latest research progress in recent years.

## 2. Sterilization Mechanism of Low Temperature Plasma

As low-temperature plasma sterilization technology is a new kind of sterilization technology, its application in food industry is not mature, and so, there is not an exact statement about its sterilization mechanism. Also, a generally convincing sterilization mechanism is not yet established. It is generally believed that the bactericidal effect of plasma is related to the charged particles (electrons, ions), active substances (molecules, excited atoms, metastable atoms, free radicals) and ultraviolet rays.

### 2.1. Ultraviolet Light

During the operation of the plasma, a large number of ultraviolet rays can be generated due to the glow discharge. Ultraviolet light can destroy the amino acid structure of proteins, or inhibit the reproduction of bacteria by interfering with the formation of thymine in the cell’s DNA. Roth et al. [23] confirmed that a certain amount of ultraviolet light will be produced in the process of plasma operation. They believed that the ultraviolet light produced is the main reason for the plasma’s bactericidal effect. Trompeter et al. [24] experimentally confirmed that UV radiation can kill Bacillus subtilis. However, the conditions for ultraviolet sterilization are that the wavelength is between 220 and 280 nm, that the sterilization effect is the best, and the light intensity is appropriate. If these conditions are not met, the ultraviolet sterilization effect will not be significant. Other scientists believe that the germicidal effect of ultraviolet light is not significant, because they are easily absorbed by gas atoms and molecules under atmospheric pressure [25].

### 2.2. Charged Particles

The charged particles produced by plasma directly act on microorganisms, which can directly destroy proteins, nucleic acids, and other macromolecular substances, leading to cell lysis and death. They will gather on the surface of cell membrane to generate strong electrostatic force, which will eventually lead to cell rupture and achieve the effect of sterilization. Therefore, some scientists believed that charged particles play a major role in the process of plasma sterilization [26]. Other scientists confirmed the damage of charged particles on cell membrane by comparing the effects of pulsed electric field and plasma on cell membrane [27], because charged particles produced by pulsed electric field can cause cell membrane perforation, and plasma also causes cell membrane perforation in a similar way. Thus, they thought that the similarity between plasma and the pulsed electric field on cell was due to the production and role of charged particles.

### 2.3. Active Ingredients

The plasma sterilization process will produce many active substances, including ozone, hydroxyl groups, nitric oxide free radicals, superoxides, etc.; they will destroy nucleic acid substances and proteins in cells, and eventually cause cell damage and even death. Guzel-Seydim et al. [28] believed that these active substances hinder the normal transmembrane transport of cells by destroying the double bonds of unsaturated fatty acids on the cell membrane, and eventually lead to cell inactivation. Some scientists thought that the active substances produced by plasma can directly pass through the cell membrane and indirectly destroy the cell structure by oxidizing the nucleic acids and proteins in the cell to achieve the purpose of sterilization [29].

## 3. Factors Affecting Sterilization Effect

### 3.1. Plasma Equipment Factors

The sterilization effect of plasma is significantly influenced by various internal factors of the plasma equipment itself. These include the type of plasma-generating device, the gas composition used as the excitation medium, the processing voltage, and the processing time. Most gas discharges can generate plasma and the sterilization effect is strongly related to the type of gas. Weng et al. [30] found that using a mixture of argon and oxygen as the excitation medium resulted in a higher sterilization rate for *Escherichia coli* compared to using a single gas. Similarly, Hury et al. [31] found that oxygen as the excitation medium exhibited a higher bactericidal rate against *B. subtilis* than pure argon. Therefore, utilizing a mixed gas as the excitation medium tends to enhance the sterilization effect compared to using a single gas. Furthermore, the optimization of processing parameters, such as treatment time, voltage, and distance, plays a crucial role in achieving effective sterilization. Sun et al. [32] treated cucumbers inoculated with *E. coli* with different treatment time, treatment voltage, and treatment distance. Within a certain range, higher treatment voltages and longer treatment times resulted in higher sterilization rates. Notably, at a treatment voltage of 170 V, a treatment time of 5 min, and a pole distance of 2.5 cm, a sterilization rate of 99.65% was achieved.

### 3.2. Reactant and Microorganism Factors

Apart from the equipment factors, the characteristics of reactants and the types and concentrations of microorganisms also play a crucial role in determining the sterilization rate. The composition and state of food, as well as the type and concentration of microorganisms present, significantly impact the effectiveness of the sterilization process. It was observed that cells in a stable phase are more susceptible to destruction than those in exponential phase. Additionally, Gram-negative bacteria tend to be more vulnerable than Gram-positive bacteria [33]. Gaunt et al. [34] conducted experiments on *Salmonella typhimurium* and *Listeria monocytogenes* with cold plasma and found that *S. typhimurium* was more easily eliminated, because the cell wall of *L. monocytogenes* is much thicker than that of *S. typhimurium*. The thickness of the cell wall affects the time required for the destruction of cells by the active substances produced by plasma. In addition, an increase in relative humidity was shown to improve the rate of plasma sterilization, as it led to an increase in the number of hydroxyl radicals and bacteria inactivation. Recently, Pan et al. [35] reported that 2 min plasma treatment after 15 min ultrasonic-assisted treatment time could lead the leakage of intracellular ROS of *L. monocytogenes*, indicating an increased susceptibility of *L. monocytogenes* cells to cold plasma treatment after ultrasound pretreatment.

## 4. Application Status of Plasma in Food Industry

### 4.1. Application of Plasma in Different Foods

#### 4.1.1. Application of Plasma in Vegetables and Fruits

From the planting to marketing of fruits and vegetables, infectious pathogenic microorganisms such as *E. coli* are attached to the surface. Therefore, in order to kill microorganisms and pests on the surface, a large number of pesticides are sprayed on the surface during planting and marketing. Pesticides will inevitably remain on the surface of fruits and vegetables and cannot be completely washed by normal water washing. Plasma sterilization of fruit and vegetable surfaces can not only effectively kill attached pathogenic microorganisms, but also degrade residual pesticides to a certain extent (Table 1).

Recently, a study showed that the number of spores inoculated on black pepper decreased by 1 log after plasma treatment, and the total phenol content of black pepper was not affected by plasma [36]. Cold plasma treatment can not only significantly reduce the amount of *Zygosaccharomyces rouxii* in apple juice, but also maintain its physical and chemical characteristics. Except for slight changes in pH and color, there was no significant change in components such as soluble solids, volatile compounds, titrated acidity, and reducing sugars [37]. Bermúdez-Aguirre et al. [38] used plasma to treat lettuce, carrots, and tomatoes that contaminated *E. coli*, and found that the sterilization effect of plasma was related to the concentration of inoculation, and the sterilization effect was better for fruits and vegetables with low bacterial concentration, after treatment, the color parameters of the vegetables did not change significantly, and the microbial cell membrane was damaged and degenerated after the treatment. Misra et al. [39] studied the effect of DBD plasma on strawberry. The microorganisms on strawberry include thermophilic aerobic bacteria and fungi (mold, yeast, etc.), and the number of microorganisms decreased to 2 log reduction within 5 min after treatment. The color and hardness of strawberry were not affected by plasma treatment. Wang et al. [40] treated blueberry fruit with DBD plasma at 45 kV for 50 s and measured the changes of the total number of bacterial colonies and quality indexes of blueberry fruit within a certain period after treatment. The results showed that the number of bacteria and fungi on the surface of blueberry decreased by 1.75 log CFU/g and 1.77 log CFU/g, respectively, and effectively delayed the decay process of blueberry, and the hardness and VC content of blueberry decreased. The result showed that the activity of antioxidant enzymes was increased and there was no effect on Anthocyanin of blueberry. Li et al. [41] treated fresh-cut pitaya fruit with plasma; the results showed that the plasma not only effectively inhibited the increase in bacteria, maintained the safety of fresh-cut pitaya fruit, but also induced the accumulation of phenol in fresh-cut dragon fruit to a certain extent, and enhanced antioxidant activity. Therefore, plasma can be used for sterilization and preservation of fresh-cut fruits and vegetables. Zhao et al. [42] compared the effects of plasma and pasteurization on the sterilization ability and sensory quality of radish *paocai*. Plasma treatment can kill aerobic yeast and yeast, and retain lactic acid bacteria. There was no significant effect on salt content and amino acid nitrogen. It can also delay the softening and browning of *paocai* during storage. Although pasteurization can almost kill all microorganisms, heat treatment accelerates the softening and browning of radish *paocai*. These results show that plasma can not only effectively sterilize, but also improve the sensory quality and storage stability of *paocai*.

Bursać Kovačević et al. [43] studied the effect of plasma on anthocyanins and color in pomegranate juice. Compared with untreated pomegranate juice, the anthocyanin content of pomegranate juice was higher after plasma treatment (21–35%). The result showed that the anthocyanin content of the samples increased with the increase in air flow and sample volume. This was due to the improved performance of anthocyanin extraction and the destruction of cell membrane integrity. Plasma treatment has a positive effect on anthocyanin stability and color change in pomegranate juice. Hou et al. [44] confirmed that compared with heat treatment, plasma treatment has less effect on the sensory quality of blueberry juice. Plasma treatment can not only significantly reduce the number of *Bacillus*, but also increase the content of phenolic substances, better maintain the original color of blueberry juice. If the treatment time is too long, the anthocyanin, vitamin C, and antioxidant activity will be reduced.

Pankaj et al. [45] studied the applicability of plasma as a new method for enzyme inactivation. They treated tomatoes with different treatment voltages and treatment times, and found that the activity of tomato peroxidase decreased with the increase in treatment time and voltage. It showed that the plasma has a certain inactivation effect on the enzyme to a certain extent, which is beneficial to the storage of fruits and vegetables. The degradation of pesticides on the surface of fruits and vegetables by plasma was also very significant. Sarangapani et al. [46] treated blueberries with DBD plasma at 80 kV for 5 min, and found that the degradation rate of imidacloprid and pyridylamine on blueberries by plasma was 75.62% and 80.18%, which did not cause physical damage to the samples and did not change the color. Zhang et al. [47] studied the feasibility of cold plasma as a non-thermal pretreatment technology for chili pepper drying. The results showed that cold plasma can improve the drying speed and anti-oxidation ability, and can effectively retain the red pigment content. However, too much treatment time may lead to the loss of pigment, so it is necessary to control the treatment time, and the best treatment time was 30 s.

Low-temperature plasma technology was widely used in the sterilization and fresh-keeping of fruits and vegetables and their products. It not only improves the microbial safety of fruits and vegetables, effectively inhibits the growth and reproduction of microorganisms during storage, but also delays the changes of physical and chemical indicators and sensory quality of fruits and vegetables during storage, and it prolongs the shelf life of fruits and vegetables to a certain extent.

#### 4.1.2. Application of Plasma in Meat Food

Low temperature plasma treatment technology was widely used in meat and meat products in recent years. It ionizes the gas to generate many active factors with bacteriostatic effects. They act on the surface of meat and meat products and kill microorganisms on the surface of meat products (Table 2).

Ulbin-Figlewicz et al. [48] used helium plasma to treat pork and beef, and the results showed that the number of microorganisms on pork and beef decreased significantly and was directly proportional to the treatment time. The total number of microorganisms on pork was reduced by 1.14–1.18 log CFU/g, the number of microorganisms on beef was reduced by 0.98–2.09 log CFU/g, and argon plasma was used in the same situation, and it was found that the treatment effect was inferior to that of helium plasma. Choi et al. [49] treated dried squid silk with low temperature plasma. After 3 min of treatment, *S. aureus*, marine bacteria, and aerobic bacteria reduced 0.9, 1.6, and 2.0 log CFU/g, respectively, and the microbial inactivation rate reached 90–99%. Except the moisture content and TBARS content, the other physical and chemical indicators were not affected by the plasma, and the physical and chemical indicators and sensory characteristics of squid silk were best when treated for 2 min. Pérez-Andrés et al. [50] studied the effect of plasma on the oxidation of lipids and proteins during storage of mackerel. After treatment with a voltage of 80 kV for 5 min, there was no effect on the fatty acid content and nutritional quality of mackerel and no lipid oxidation occurred. However, after treatment, the formation of carbonyl group would be accelerated, which will accelerate the oxidation of proteins. Therefore, it is necessary to further study the influence of plasma on the quality of seafood products. Kim et al. [51] evaluated the safety of treating pork tenderloin with DBD plasma. They used a helium and oxygen mixture as discharge gas, respectively. After a 10 min treatment, *E. coli* decreased by 0.34 and 0.55 log CFU/g, while *L. monocytogenes* decreased by 0.43 and 0.59 log CFU/g. The pH and brightness of the samples decreased after treatment and the degree of lipid oxidation was not obvious. Although this method has some limitations, it is still a potential sterilization method.

Nitrite is an additive commonly used in cured meat products, but chemically synthesized nitrite reacts with secondary amines in meat products to form carcinogens, which will affect people’s health after eating. In recent years, it was confirmed that the interaction between low-temperature plasma and water medium will produce a variety of nitrides, including nitrites [57], which can be used as additives for cured meat. Jung et al. [52] compared the quality of emulsified sausages cured by PTW (plasma-treated water), nitrite-containing celery flour, and sodium nitrite at a concentration of 70 mg/kg. The results showed that PTW contained enough nitrite to pickle emulsified sausages, and the total aerobic bacteria number, color, and peroxide value of pickled sausages were similar to those pickled by sodium nitrite. In addition, the sensory acceptance of sausages cured with PTW were better than sausages cured with sodium nitrite or nitrite-containing celery flour, indicating that PTW can be used as a source of nitrite for cured meat. Yadav et al. [53] studied the effects of temperature, NaCl (%), and rosemary extract on the treatment of ready-to-eat ham by plasma. The results showed that the number of *L. innocua* on the ready-to-eat ham decreased significantly, the NaCl (%), and temperature had no significant effect on plasma treatment. However, the MDA of the treated samples increased and the water content decreased. Therefore, the drying and oxidation of ham should be performed under open atmospheric plasma treatment conditions. Gök et al. [54] showed that the treatment of dry cured beef products with cold plasma can change the water activity, water content, and TBARS value, but it can reduce the number of *S. aureus* and *L. monocytogenes* by 0.85 log CFU/cm^2^ and 0.83 log CFU/cm^2^, and aerobic bacteria and yeast–mold counts were also significantly reduced. Therefore, in order to widely use this technology in the food industry, it is necessary to select processing conditions and parameters according to the quality and sensory characteristics of the product. Yong et al. [55] evaluated the bactericidal effect of plasma treatment on beef jerky and its effects on physical and chemical indicators. The results showed that plasma treatment greatly reduced the number of bacteria and mold on beef jerky. There was no change in the cutting force and myoglobin content, but the flavor of the beef jerky slightly changed. Therefore, we can further study how to minimize the change in flavor. Jung et al. [56] installed a plasma on the upper part of the meat mixer. After the system was operated, the generated plasma and the liquid in the meat emulsion in the mixer formed nitrite and the peroxide value of the meat emulsion did not change. After treatment, the meat mince presented a unique red color. Interestingly, the treatment did not affect the total aerobic bacteria in the meat batter samples, possibly due to the concurrent plasma treatment and mixing process, where the exposure time of the meat batter to plasma may not have been enough to eliminate microorganisms.

In addition, Leipold et al. [58] studied the decontamination effect of rotary cutting tools for meat slices. After 340 s of plasma treatment, the number of L. monocytogenes inoculated on the cutter was reduced by 5 log CFU/g, which can decontaminate during cutter slicing and reduce the risk of cross contamination between different batches of meat.

Low-temperature plasma treatment can not only clean the microorganisms on the surface of meat, but also generate natural nitrite to replace the addition of NaNO_2_ in the processing of cured meat. However, there are still some problems that were not solved. Firstly, because the penetration depth of the plasma is not large, the sterilization effect is not good enough for the bacteria inside the meat. Secondly, the highly active ROS produced by the low-temperature plasma can promote the oxidation of lipids in meat, and, finally, may have adverse effects on the flavor of meat. Therefore, in the future, efforts should be made to develop low-temperature plasma source suitable for meat industrial application or to combine other non-heat treatment technologies with plasma to improve its bactericidal efficiency. In order to prevent the effect of low-temperature plasma treatment on meat quality, natural antioxidants were added to inhibit the oxidation of lipids.

#### 4.1.3. Application of Plasma in Grain Industry

Low temperature plasma can not only modify the surface of starch, but also effectively shorten the cooking time of the grain and improve the performance of the grain after cooking. Therefore, plasma has a wide application prospect in the field of grain crop processing (Table 3).

The plasma treatment can improve the characteristics of grain flour to a certain extent. Misra et al. [59] treated hard and soft wheat flour with cold plasma, studied the rheological properties of wheat flour, and found that the dough properties and optimal mixing time of wheat flour were improved. The elasticity and viscosity of hard wheat flour gradually increased with the increase in processing voltage and time. Low temperature plasma can effectively improve the functional properties of wheat flour. Thirumdas et al. [60] confirmed that cold plasma treatment can change the structure, function, and rheology of natural rice starch. Plasma etching, depolymerization, and cross linking are the main mechanisms for improving starch performance by plasma.

Most grains have a hard fiber outer layer, which leads to long cooking time. Plasma technology can effectively shorten the cooking time of grains. Sarangapani et al. [61] studied the effect of low-pressure plasma treatment on cooking and texture characteristics of steamed rice at different time and power. The result showed that the water absorption rate of steamed rice after heating would increase and the cooking time could by shortening 8 min, the texture characteristics will also be improved. The plasma treatment not only maintains the high nutritional value of steamed rice, but also guarantees better quality and reduced cooking time. Potluri et al. [62] used cold plasma to treat bamboo rice under different conditions. The soaking rate of bamboo rice treated with plasma was increased by 15% and the cooking time was shortened by about 12 min. The final viscosity (306 cp) of bamboo rice treated by plasma was lower than that of untreated rice, which was caused by the degree of starch cross-linking.

In addition, plasma can also effectively improve the germination rate and other characteristics of crop seeds. Chen et al. [63] treated brown rice with a low-power plasma at 1–3 kV for 10 min, and found that the germination rate, seedling length, and water absorption of brown rice would increase after plasma treatment. The surface of brown rice may be etched by plasma treatment. As a result, the water absorption rate of brown rice increased and the germination rate also increased. The α-amylase activity of brown rice in the plasma treatment group was significantly higher than that of the control group, which was one of the reasons that caused the rapid germination of seedlings. In addition, the γ-aminobutyric acid level and antioxidant activity of the experimental group also increased significantly. Therefore, brown rice treated with plasma can make brown rice more nutritious. Yodpitak et al. [64] studied the changes in the bioactive substances of Thai germinated brown rice (GBR) after plasma treatment. The results showed that the germination rate, root length, and seedling height of brown rice increased by 84%, 57%, and 69%. Plasma can be used to promote the germination of rice seeds. Sadhu et al. showed that cold plasma treatment was beneficial to the germination of mung bean seeds under drought conditions. The germination rate, root length, electrical conductivity, and hydrophilicity of seeds after plasma treatment were significantly improved, which could promote the germination of seeds under drought conditions.

Jiang et al. [65] studied the effect of cold helium plasma on wheat seed germination, growth, and yield, and the results showed that the treatment with 80 W power can significantly improve the germination and germination rate of wheat seeds. After field experiments, compared with the control group, the seeds of the experimental group grew significantly faster, and the chlorophyll content, nitrogen content, and water content were significantly higher than the control group, which indicates that the plasma can promote the growth of wheat to a certain extent. Although it is difficult for bacteria to enter the inside of the grain of the grain, if the grain is damaged, it will cause bacterial contamination, and the plasma can effectively remove bacteria on the surface of the grain. Sadhu et al. [66] showed that cold plasma treatment was beneficial to the germination of mung bean seeds under drought conditions. The germination rate, root length, electrical conductivity, and hydrophilicity of seeds after plasma treatment were significantly improved, which could promote the germination of seeds under drought conditions.

Lee et al. [67] found that after treating brown rice with plasma for a period of time, the amount of *Bacillus cereus*, *B. subtilis*, and *E. coli* on the surface of brown rice was reduced by about 2.30 log CFU/g, and α-amylase activity and water absorption were significantly increased, and the decrease in the hardness of brown rice indicates that low-temperature plasma treatment can not only improve the microbial quality of brown rice, but also improve its physical and chemical properties. Tolouie et al. [68] confirmed that plasma treatment could lead to the inactivation of lipase and lipoxygenase, thus prolonging the shelf life of wheat germ. Although the enzyme activity recovered during storage, it was also much lower than the control group. Therefore, plasma can be used to improve the stability of wheat germ and extend the shelf life.

Low-temperature plasma plays an important role in grain processing and storage, It is not only suitable for surface purification, but also has good application prospects in removing biotoxins, modified starch, improving grain quality, removing agricultural residues, seed germination, and extending shelf life, which has a positive impact on the characteristics of grain, and has no adverse impact on the sensory quality and nutritional composition of grain.

#### 4.1.4. Application of Plasma in Dairy Products

Heat treatment can effectively kill the pathogenic bacteria in milk, but they will greatly reduce the sensory quality, nutrition, and physical and chemical properties of milk, leading to non-enzymatic browning, loss of vitamins, and volatile flavor compounds (Table 4). The lowering of the freezing point of milk eventually leads to a change in the flavor of milk [69]. Plasma technology is one of the potential new sterilization technologies in the dairy industry. Many researchers used plasma for sterilization of milk and dairy products, and verified its purification ability to milk and dairy products. Plasma could affect the structural and physical properties of bovine serum albumin by the high energetic and oxidizing species [70].

Gurol et al. [71] evaluated the purification ability of low-temperature plasma on *E. coli* in milk with different fat contents. After plasma treatment, the number of *E. coli* in fresh milk can be reduced by more than three times, and the physical and chemical properties of fresh milk were almost unchanged. The treated samples had no bacterial growth within one-week storage period. Kim et al. [72] treated fresh milk samples inoculated with *E. coli*, *L. monocytogenes*, and *S. typhimurium* with plasma treatment and found that the number of bacteria decreased significantly after 5 and 10 min, and the pH of milk slightly decreased. The characteristic values of milk L* and b* increased, the value of a* decreased, and the content of malondialdehyde produced by lipid oxidation increased slightly, but not significantly. The result showed that the treatment of milk with plasma for too long will lead to the physical and chemical indicators slight changes of milk. Korachi et al. [73] determined the biochemical changes of protein, free fatty acid, and volatile matter in fresh milk after plasma treatment. The result showed that there was no significant change in lipid composition, total ketone, and alcohol content after plasma treatment, but the long treatment time would lead to a increase in total aldehyde content in the sample. Coutinho et al. [74] studied the effect of low-pressure cold plasma treatment on the physical and chemical indexes of chocolate milk. The results confirmed that the selection of appropriate process parameters can improve the physical and chemical indexes and sensory quality of chocolate milk. If the treatment conditions were not appropriate, it would lead to a decrease in biological compounds and volatile compounds, the change of fatty acid composition, and other adverse effects. Therefore, it is very important to select the appropriate process parameters. Chen et al. [75] evaluated the bactericidal effect of cold atmospheric plasma on *E. sakazakii* in non-fat dry milk (NFDM). After 120 s of treatment, *E. sakazakii* could reduce 3.27 log CFU/g and had no effect on the physical and chemical indexes of NFDM. Lee et al. [76] treated the cheese inoculated with *E. coli* and *S. aureus* with plasma, and also evaluated the color parameters and sensory quality of sliced cheese after plasma treatment at different times. The results showed that the number of *E. coli* and *S. aureus* decreased significantly. The characteristic value L* decreased significantly, and the value of b* increased. After treatment for 15 min, the sensory quality of cheese slices decreased significantly, including flavor and smell. The result showed that the quality of food is closely related to the treatment time of plasma. Wan et al. [77] studied the bactericidal effect of high-pressure atmospheric cold plasma (HVACP) on Queso Fresco cheese (QFC) and cheese model (CM). After 5 min of treatment, the *Listeria* on TSA, CM, and QFC decreased 5.0, 3.5, 1.6 log CFU/g, respectively. This was due to the different microstructure and roughness of the substrate. The smoother the surface of the substrate, the better the sterilization effect. Therefore, different processing parameters need to be selected according to the surface structure of the substrate. Segat et al. [78] treated whey protein isolate (WPI) with atmospheric cold plasma. After treatment, the pH value decreased and the sample color began to turn yellow. After 15 min of treatment, the protein oxidized. After 60 min treatment, HPLC and DLS showed that plasma treatment improved the foaming and emulsifying capacity of whey protein isolate, and the average particle size, dispersion index, and foam stability increased. Plasma can be successfully applied to selectively modify protein structure, thereby improving WPI function.

Low-temperature plasma can improve the microbial safety of milk and dairy products to a certain extent, but for substrates with uneven surface morphology such as cheese, the sterilization effect of plasma will be relatively reduced. Plasma treatment has lower requirements on the environment and is usually operated at normal temperature, so it has less impact on the substrate. However, if the treatment conditions are not appropriate or the treatment time is too long, it will accelerate the lipid oxidation and adversely affect the sensory quality. Therefore, further exploration is needed to reduce its negative impact.

### 4.2. Application of Plasma in Other Ways

#### 4.2.1. Application of Plasma in Food Packaging

Food packaging can not only guarantee the functional characteristics of food, but also prevent the food from being contaminated and damaged by microorganisms during transportation and storage. If the storage conditions are not suitable, the packaging materials may be contaminated by microorganisms, which, in turn, will contaminate the food, eventually leading to food spoilage and economic loss [79]. Packaging surface treatment includes surface functionalization, surface cleaning, etc. Plasma can be used for the surface treatment of packaging materials. It can not only effectively kill microorganisms on the surface of packaging, but also effectively improve the characteristics of packaging.

Lei et al. [80] studied the effect of atmospheric pressure plasma on polyethylene terephthalate/polypropylene film. After plasma treatment, the surface hydrophilicity and roughness of the film increased. After treatment, the inhibition rate of the film to *B. subtilis* and *E. coli* reached almost 100%, and the inhibition rate to *S. aureus* was less than 85%. Lee et al. [81] studied the decontamination ability of low-pressure glow discharge plasma on the surface of ordinary food packaging materials (glass, polyethylene, polypropylene, nylon, paper foil, etc.), and within a certain range of vacuum pressure and power density, the optical properties, color characteristics, surface temperature, tensile strength of the packaging surface after plasma treatment had no significant change, and they were under treatment within 5 min, the number of pathogenic bacteria, especially *E. coli* and *S. aureus*, was greatly reduced. Oh et al. [82] treated the edible film made from defatted soybean meal of smoked salmon with cold plasma under the condition of 400 W treatment voltage and 15 min treatment time. The elongation and water barrier of the film increased greatly after treatment. Pankaj et al. [83] used atomic force microscope (AFM) to observe the surface morphology of zein film. The roughness of zein film after plasma treatment increased from 20 nm to 100 nm, which was due to the surface corrosion during the plasma treatment. Wu et al. [84] successfully improved the packaging properties of casein edible film with DBD plasma, including its mechanical properties and barrier properties. In the process of plasma treatment, crystal migration and protein aggregation took place in the film, which enhanced the stability of the film structure. Food packaging plays a crucial role in ensuring the quality and safety of food products by preventing contamination and damage during transportation and storage. Studies demonstrated the positive effects of plasma treatment on various packaging materials. Atmospheric pressure plasma treatment increased the surface hydrophilicity and roughness of polyethylene terephthalate/polypropylene films, resulting in a significant inhibition in bacterial growth. Low-pressure glow discharge plasma effectively decontaminated the surfaces of common food packaging materials without compromising their optical properties, color characteristics, and mechanical strength. Plasma treatment also improved the properties of edible films made from defatted soybean meal and casein, enhancing their water barrier, mechanical strength, and stability.

Low temperature plasma technology was used in food packaging materials processing and modification research. Through the surface modification of food packaging materials, the surface energy and water contact angle were reduced, and the air permeability, water permeability, thermoplastic and thermal stability of packaging materials were improved. At present, food sterilization mostly adopts the way of first sterilization and then packaging. Due to the secondary pollution in the packaging process, the growth and propagation of contaminated microorganisms lead to food corruption and deterioration in the package. However, there is relatively little research on the food sterilization method of first packaging and then sterilization.

#### 4.2.2. Application of Plasma-Activated Water

After water was treated with plasma, the redox potential and conductivity changed, the reactive oxygen species (ROS) and nitrogen species (RNS) were also generated, which eventually caused the chemical composition of water to change [85]. This water is called plasma-activated water (PAW). In recent years, PAW was widely used in the food industry.

Liao et al. [86] compared the preservation effect of TW ice and PAW ice on fresh shrimps, and the results confirmed that PAW ice not only inhibited microbial growth during storage, but also delayed changes in color and hardness, and extended the shelf life of fresh shrimps by 4–8 day. Xiang et al. [87] used PAW to clean mung bean sprouts and found that PAW can not only greatly reduce the number of bacteria, yeasts, and molds on mung bean sprouts, but also had no adverse effect on the physical and chemical indicators and sensory quality of mung bean sprouts. Reactive species such as nitrate, nitrite, and H_2_O_2_ produced in PAW may enhance the disinfection effect of PAW. In addition, PAW has excellent fresh-keeping effects on fresh-cut fruits and vegetables. Fresh-cut “Fuji” apples were immersed in PAW for 5 min and stored at 4 ± 1 °C. PAW effectively inhibited the growth of microorganisms. The total number of colonies on fresh-cut apples after storage for 12 days did not exceed 5 log CFU/g. PAW also inhibited the occurrence of browning and had no effect on the hardness and titratable acidity of apples [88]. Liao et al. [89] used PAW as the thawing media and studied its influence on the microbial safety and sensory quality of beef. During thawing, PAW effectively reduced the number of bacteria, fungi, and yeast on beef. Compared with traditional thawing medias, PAW did not have any adverse effects on the texture, pH, and color of beef, and it better inhibited the oxidation of lipids and proteins in beef. Studies demonstrated the efficacy of PAW in various applications. For instance, PAW ice was found to inhibit microbial growth and delay changes in color and texture, leading to an extended shelf life of fresh shrimps. PAW was also effective in reducing bacterial, yeast, and mold populations on mung bean sprouts without affecting their physical and chemical indicators or sensory quality.

In addition, PAW can be used in combination with other physical methods to improve the microbial safety of food. When PAW was used in combination with mild heat (60 °C, 4 min), the amount of *E. coli* decreased from 8.28 log CFU/mL to an undetectable level, and the bactericidal effect was significantly higher than using PAW alone or mild heat. The combination of the two methods increased the membrane permeability of *E. coli* cells, led to the leakage of proteins, nucleic acids, and other substances in the cells, and finally led to the inactivation of *E. coli* cells [90]. Sequential combination of washing with 120-PAW followed by mild heat, the amount of *L. monocytogenes* and *S. aureus* inoculated on salted Chinese cabbage decreased by 3.4 and 3.7 log CFU/g, respectively. This combined treatment did not reduce the quality of pickled salted Chinese cabbage, but reduced the salinity and peroxidase activity to a certain extent [91]. Lo Porto et al. [92] studied the effect of the combination of PAW and ultrasound on the germination and growth of soybean. The results showed that the combination of PAW and ultrasound not only improved the germination time of the seed, but also enhanced the hydration and the ability to absorb water of the seed.

These findings highlight the potential of PAW as a versatile and sustainable solution for improving food safety and quality. As a new and environmental protection treatment technology, PAW has many advantages, such as wide range of treatment, no secondary pollution, and so on. It has a broad application prospect in the food processing and safety fields, such as fruit and vegetable sterilization and preservation, meat products preservation and color protection, and so on. After that, we should focus on the application of PAW in the fields of food raw materials sterilization and fresh-keeping, food contact surface disinfection, and evaluate its impact on the physical and chemical indexes and sensory quality of food, to promote the wide application of PAW in the food industry.

## 5. Conclusions

Low temperature plasma technology is a unique and effective non-thermal sterilization technology, which can hardly damage the nutritional components and sensory quality of food, and it also has the advantages of fast, safe, efficient, environmentally friendly, simple operation, and no toxic substances. In recent years, plasma was widely used in the food industry. It is mainly used for sterilization and freshness of various foods, which not only extends the shelf life of food, but also improves the performance of food to a certain extent, so it can be used as a potential new technology that can replace traditional sterilization methods.

## Figures and Tables

**Table 1:** The effect of low temperature plasma on vegetables and fruits.

Food Matrix	Conditions	Results	References
black pepper	voltages of 15 and 30 kV for 3–20 min	Up to 2 log and 1 log reduction in *Bacillus subtilis* vegetative cells and spores achieved, respectively	[36]
apple juice	voltages of 21 kV for 30 min	5.6 log reduction in yeast (*Zygosaccharomyces rouxii*) reached	[37]
lettuce, carrots and tomatoes	3.95 kV up to 12.83 kV (60 Hz) in argon, from 30 s to 10 min	the highest voltage and longest treatment time could reach 1.6 log reduction in pathogenic *Escherichia coli*	[38]
strawberries	60 kV for 5 min	2 log reduction in the background microflora (aerobic mesophilic bacteria, yeast, and mold) achieved	[39]
blueberries	45 kV for 50 s	Total aerobic mesophilic bacteria and yeast/mold counts were decreased by 1.75 and 1.77 log reduction	[40]
fresh-cut pitaya	60 kV for 5 min	significantly inhibited the growth of total aerobic bacterial counts, increased the cutting-induced phenolic accumulation, and enhanced antioxidant activity in fresh-cut pitaya fruit	[41]
fermented vegetable (radish paocai)	60 kV for 60 s	efficiently eliminated yeasts, especially gas-producing yeast	[42]
pomegranate juice	5 cm^3^ sample volume, and 0.75 dm^3^/min gas flow at 6 W for 3 min	Reached the greatest anthocyanin stability	[43]
blueberry juice	11 kV for 4 min	Significantly increased the content of phenolics and better kept the original color	[44]
tomato	30, 40 and 50 kV for different time	the activity of tomato peroxidase decreased with the increase in treatment time and volt-age	[45]
blueberries	80 kV for 5 min	The degradation efficacy of pesticides of 80.18% for boscalid and 75.62% for Imidacloprid reached, respectively,	[46]
chili pepper	750 W for 15, 30, 45, and 60 s	improve the drying speed and anti-oxidation ability, and can effectively retain the red pigment content	[47]

**Table 2:** The effect of low temperature plasma on meat products.

Food Matrix	Conditions	Results	References
Pork and beef	21 kV for 10 min	the total number of microorganisms, yeasts, and molds, and psychrotrophic microorganisms was reduced in the range of 1.14–1.48 log cycles for pork and 0.98–2.09 log cycles for beef	[48]
dried squid shreds	20 kV for 0–3 min	aerobic bacteria, marine bacteria, and *Staphylococcus aureus* were inactivated by 2.0, 1.6, and 0.9 log units, respectively.	[49]
mackerel	80 kV for 5 min	no significant changes were found in lipid oxidation, as well as the fatty acid composition or nutritional quality indices after treatment	[50]
pork loin	3 kV for 5 and 10 min	*E. coli* was reduced by 0.26 and 0.55 log cycles, while *Listeria monocytogenes* was reduced from 0.17 to 0.59 log cycles	[51]
sausage	10 W/cm^2^	there were no noticeable effects on the total aerobic bacterial counts, color, and peroxide values of sausages	[52]
ready-to-eat ham	300 W for 3 min	a significant reduction in *L. innocua* of 1.51 to 1.75 log CFU/cm^2^ at 4 °C, while 1.43 to 1.78 log CFU/cm^2^ at 23 °C	[53]
dry-cured beef product	25 kV for 5 min	Maximum reduction of 0.85 log CFU/cm^2^ for *S. aureus* and 0.83 log CFU/cm^2^ for *L. monocytogenes*, while 1.41 log CFU/cm^2^ for aerobic bacteria and 1.66 log CFU/cm^2^ for yeast–mold counts, respectively	[54]
beef jerky	flexible thin-layer plasma 10 min treatment	*E. coli* O157:H7, *L. monocytogenes*, *Salmonella Typhimurium*, and Aspergillus flavus were reduced by approximately 2 to 3 log CFU/g	[55]
meat batter	550 W 30 min	Total aerobic bacterial count of meat batter was not influenced and the nitrite level increased to 65.96 ppm	[56]

**Table 3:** The effect of low temperature plasma on grain crop products.

Food Matrix	Conditions	Results	References
wheat flour	voltages of 60 and 70 kV for 5 and 10 min.	an improvement in the dough strength and optimum mixing time for both strong and weak wheat flours.	[59]
rice starch	two different power levels 40 W and 60 W for 5 and 10 min	change the structure, function, and rheology of natural rice starch	[60]
rice flour	at varying power of 30 W, 40 W and 50 W for duration of 5, 10 and 15 min	the water absorption rate of steamed rice after heating would increase and the cooking time could by shortening 8 min, the texture characteristics will also be improved	[61]
bamboo rice	15, 20, and 25 W/cm^2^ for 5 and 10 min	The soaking rate of bamboo rice was increased by 15% and the cooking time was shortened by about 12 min	[62]
brown rice	ranging from 1 to 3 kV for 10 min	the germination rate, seedling length, and water absorption of brown rice would increase	[63]
Thai germinated brown rice	100–200 W for 75 s	the germination percentage, root length, and seedling height measurements of the most sensitive rice cultivar increased by 84%, 57%, and 69%, respectively	[64]
Wheat	60 W, 80 W and 100 W for 15 s	improve seed germination potential (6.0%) and germination rate (6.7%)	[65]
mung beans	two different power levels 40 W and 60 W for 10, 15 and 20 min	increased the germination rate by 36.2%, radical root length by 20% and conductivity of seeds by 102%	[66]
brown rice	250 W for periods of 5, 10 and 20 min	a 20 min plasma treatment resulted in a reduction in bacterial counts by approximately 2.30 log CFU/g	[67]
wheat germs	voltages of 20 and 24 kV for 5–35 min	25 min ACP treatment resulted in reduction in lipase and lipoxygenase activity of WG to 25.03% and 49.98% of initial extent, respectively.	[68]

**Table 4:** The effect of low temperature plasma on dairy products.

Food Matrix	Conditions	Results	References
milk	9 kV for 3, 6, 9, 12, 15 and 20 min	4.15 log CFU/mL *E. coli* in whole milk decreased; did not cause any significant change to the pH and color values	[71]
milk	250 W for 5 and 10 min	Total aerobic bacterial count (0.98 log CFU/mL) was eliminated. Approximately 2.40 log CFU/mL decrease in *Escherichia coli*, *Listeria monocytogenes*, and *Salmonella Typhimurium* achieved	[72]
milk	9 kV for 20 min	Significantly increased the total aldehyde content. No significant difference was observed in the total ketone or alcohol levels	[73]
chocolate milk	400 W at gas flow rates of 10, 20, and 30 mL/min for 5, 10, and 15 min	Different treatment condition showed different effect on physio-chemical characteristics, bioactive compounds, fatty acid composition, and volatile compounds profile of chocolate milk drink	[74]
milk powder	4.4 kV for 20–120 s	Led to 1.17–3.27 log10 reductions in *Cronobacter sakazakii*	[75]
Cheese Slices	3.5 kV for 1, 5, 10 and 15 min	0.09–1.47 log CFU/g and 0.05–1.98 log CFU/g decrease in *E. coli* with helium and He/O_2_, while 0.05 to 0.45 log CFU/g and 0.08 to 0.91 log CFU/g decrease in *S. aureus*	[76]
cheese	100 kV for 5 min	1.6 log CFU/g decrease in *Listeria innocua* achieved	[77]
Whey protein isolate	70 kV for 1, 5, 10, 15, 30 and 60 min	an increase in carbonyl groups and the surface hydrophobicity, while the reduction in free SH groups indicated mild oxidation occurred in the proteins	[78]

## Data Availability

Not applicable.
